# Behavioral Interactions Shaping the Use of a Mobile Health Intervention for Diabetic Foot Self-Care Among People With Diabetes and Caregivers: COM-B and TDF Qualitative Analysis

**DOI:** 10.2196/101195

**Published:** 2026-11-10

**Authors:** Sofia Malena Rebull, Guillermo Almeida-Huanca, Josselyn Jauregui Mantilla, Lourdes Cruzado-Castro, Sebastian García Cruz, Pablo Perel, Juan Jaime Miranda, Juned Siddique, Jacqueline Seiglie, Hueiming Liu, Stephen Jan, Laura Downey, David Beran, David G Armstrong, German Comina, María Lazo-Porras, Jessica Hanae Zafra-Tanaka

**Affiliations:** 1 CRONICAS Center of Excellence in Chronic Diseases, Universidad Peruana Cayetano Heredia, Av. Armendariz 445, Miraflores, Lima, Lima Province, 15074, Peru, 51 1 241 6978; 2 Faculty of Epidemiology and Population Health, London School of Hygiene & Tropical Medicine, London, England, United Kingdom; 3 Sydney School of Public Health, Faculty of Medicine and Health, The University of Sydney, Sydney, New South Wales, Australia; 4 Feinberg School of Medicine, Northwestern University, Chicago, IL, United States; 5 Department of Medicine, Massachusetts General Hospital, Boston, MA, United States; 6 The George Institute for Global Health, UNSW Sydney, Sydney, New South Wales, Australia; 7 The George Institute for Global Health, Imperial College London, London, England, United Kingdom; 8 Division of Tropical and Humanitarian Medicine, Faculty of Medicine, University of Geneva and Geneva University Hospitals, Geneva, Switzerland; 9 Department of Surgery, Keck School of Medicine, University of Southern California, Los Angeles, CA, United States; 10 Research Group in Engineering for Health and the Environment (SAMA-UNI), National University of Engineering Peru, Lima, Lima Province, Peru

**Keywords:** diabetic foot, TDF, mobile health intervention, AI, low- and middle-income countries, behavioral change, facilitators, barriers, COM-B model

## Abstract

**Background:**

Mobile health (mHealth) interventions show promise for supporting diabetic foot self-care; however, sustained engagement remains a challenge, particularly in low- and middle-income countries. While educational strategies are commonly used to address diabetic foot ulcers, they often fail to translate knowledge into long-term self-care. Understanding behavioral mechanisms underlying mHealth use, including the role of caregivers, is critical for improving implementation and effectiveness.

**Objective:**

This study explored how behavioral interactions between people with diabetes and caregivers shape the use of an mHealth app for diabetic foot self-care.

**Methods:**

This qualitative explanatory study was embedded within a parent study of an mHealth app for diabetic foot care that was conducted in primary care settings in Peru. It used the COM-B (capability, opportunity, motivation–behavior) model and the Theoretical Domains Framework (TDF) to identify mechanisms relevant for intervention design and scale-up. Semistructured interviews were conducted with people with diabetes and caregivers about their experiences related to accessing the app. Data were analyzed using deductive and inductive thematic analysis informed by the COM-B and TDF.

**Results:**

In total, 14 people with diabetes (median age 54 y, 8 women and 6 men) and 10 caregivers (median age 28.5 y, all women) were interviewed. The app use depended on varying interactions between COM-B domains, while other behavioral determinants aligned with a single domain. Across domains, interactions between people with diabetes and caregivers also affected the app use. For example, caregivers often compensated for physical capability limitations through foot positioning or environmental adjustments. Environmental context and resources, including internet access, lighting, and device compatibility, affected the app use for all participants. Motivation for all participants was largely driven by beliefs about the app’s preventive value, although reassurance from health professionals and altruism also emerged.

**Conclusions:**

The app use often depended on collaboration between people with diabetes and caregivers as a dyad, rather than on independent users. This highlights the need to design digital interventions around caregiver demographics, competing responsibilities, and the additional responsibilities an app may introduce. Participants’ preference for individual follow-up suggests that nonphysician health personnel and other human support may help maintain continuity and reassurance alongside digital monitoring. Although the app was an early prototype intended for algorithmic training, participant experiences provided insight into real-world usability and contextual barriers for future app development.

## Introduction

It is estimated that up to one-third of people with diabetes will develop a diabetic foot ulcer (DFU) in their lifetime [1]. DFU remains one of the most severe and preventable complications of diabetes, requiring continuous self-care behaviors such as routine foot inspection and early detection of preulcerative lesions [1,2]. Mobile health (mHealth) technologies have emerged as promising tools to support these behaviors by enabling remote monitoring, patient education, and self-management support [3]. Systematic reviews suggest that mobile app–assisted interventions can improve diabetes management outcomes and facilitate self-care behaviors; however, sustained engagement with these technologies remains inconsistent in real-world settings [4,5]. Many interventions report declining use over time, indicating that the availability of digital tools alone is insufficient to ensure long-term behavioral adoption [6-8].

Caregivers frequently play a critical role in supporting diabetes self-management, particularly in low- and middle-income countries, where family members often assist with daily health tasks, monitoring, and decision-making [9-11]. For individuals who are older or have mobility limitations, visual impairments, or lower digital literacy, caregiver involvement may become essential for technological support and the navigation of app interfaces. Despite this reality, most digital health interventions conceptualize technology use as an individual behavior, rarely examining caregivers as explicit behavioral actors influencing adoption and sustained use.

Previous studies on mHealth interventions for diabetic foot prevention have primarily focused on clinical outcomes, technological performance, or usability assessments [12,13]. While these studies provide important evidence on feasibility and acceptability, they often overlook the behavioral mechanisms that determine whether individuals incorporate digital tools into everyday routines. Moreover, the factors that influence the behavior of caregivers of people with diabetes have not been explored in this context. As a result, there remains a limited understanding of how sustained engagement with mHealth technologies for diabetic foot self-monitoring is influenced by personal, physical, and social environments [7].

To better understand and improve sustained engagement with these types of interventions, behavioral theory can provide a structured framework for examining determinants of technology use. The COM-B (capability, opportunity, motivation–behavior) model conceptualizes behavior as the result of interactions between individuals’ capabilities, opportunities, and motivations [14]. The Theoretical Domains Framework (TDF) complements COM-B by providing additional granularity to explore underlying mechanisms, such as knowledge, skills, social influences, reinforcement, and emotional responses, which influence behavioral performance [15]. Together, these frameworks enable a systematic analysis of how behavioral determinants interact within real-world contexts to shape the engagement of people with diabetes and caregivers with digital health interventions.

## Methods

### Study Context

The Foot Selfie study was established as an innovative approach to address the burden of DFU in Peru, emphasizing prevention and early identification of foot ulcers. In phase 1 of the project, an initial version of a mobile app (Android package kit [APK]) was designed with a single functionality of capturing and uploading foot images for training an AI algorithm to detect healthy skin and 6 preulcerative lesions. These lesions included fissures, erythemas, calluses, hemorrhagic calluses, discoloration, and ulcers. The first cohort of participants for image collection included 1000 adults with type 1 or type 2 diabetes mellitus in hospital settings. After initial image collection in hospital settings, a subsample of up to 200 individuals was invited, according to specific selection criteria, to use the app at home for follow-up image capture. In total, 12,164 images were collected from phase 1. During phase 2 of the parent study, individuals who participated in at-home follow-up were selected to participate in this study according to the selection criteria detailed below. At this stage, the app was designed to be fully self-directed and did not provide diagnostic feedback. Participants were informed that submitted images were not a substitute for clinical care. However, if the research team identified a concerning lesion or potential complication during study procedures, participants were contacted and advised to seek appropriate clinical evaluation. Weekly follow-up from field personnel (nurses) emerged somewhat organically and typically occurred when participants stopped using the app.

### Study Design

This study used a qualitative descriptive design and was explicitly informed by the COM-B model to examine determinants of app use behavior [14]. To deepen interpretation, domains from the TDF were used to provide additional context within the COM-B domains [16] (Figure 1). The target behavior for all participants was defined as the use of the app to capture photographs of the foot for early detection of preulcerative lesions.

People with type 2 diabetes and caregivers who had participated in at-home use of the early prototype for a 1-month period and had consented to be recontacted were invited to participate in semistructured interviews. Data were collected between August 2024 and February 2025. The COREQ (Consolidated Criteria for Reporting Qualitative Research) was used to ensure detailed reporting [17].

**Figure 1.**
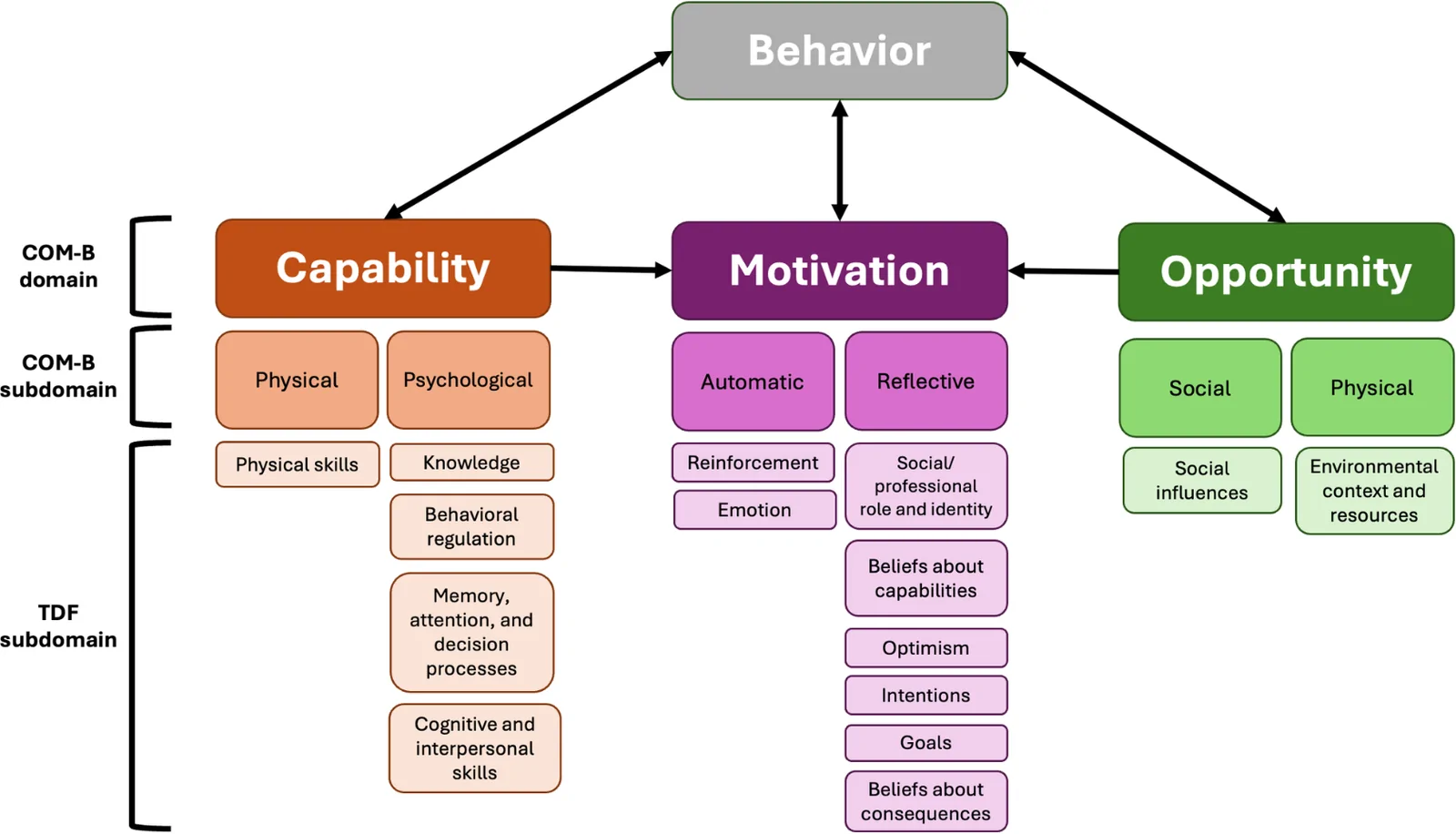
Overview of the COM-B (capability, opportunity, motivation–behavior) model and Theoretical Domains Framework (TDF). This figure demonstrates the 3 COM-B domains, their subdomains, and the associated TDF domains that inform behavior. Adapted from Atkins et al [16]. This framework was applied independently to each participant—people with diabetes and caregivers—and compared afterward.

### Study Setting

Qualitative data collection was conducted in Lima, Peru, a region selected for its epidemiological relevance to the burden of diabetes. Lima, the country’s capital, concentrates approximately one-third of the national population and reported the highest number of diabetes cases in 2025 [18].

The people with diabetes in the study were receiving care for this condition at endocrinology and diabetic foot service units at 5 high-complexity (category III-1, according to the Peruvian Ministry of Health classification) hospitals: Hospital María Auxiliadora, Hospital Dos de Mayo, Hospital Cayetano Heredia, Hospital Arzobispo Loayza, and Hospital de Emergencias Villa El Salvador.

The multidisciplinary research team was composed of researchers with backgrounds in public health, social sciences, and medicine, with significant collective experience in diabetes research. Interview supervision and data collection were led by an anthropologist with extensive expertise in qualitative research and behavioral design.

All interviewers had prior training in qualitative interviewing, and they did not have an established personal relationship with participants at the time the interviews were conducted.

The analysis was supported by team members trained in public health, biology, and project management and was led by a researcher with a background in epidemiology and global health. Discussions were held throughout the study to consider how researchers’ backgrounds could influence data collection and interpretation.

### Study Participants and Sampling

For interview selection, a purposive sampling strategy with predefined user profiles was used to ensure variation in experiences with app use. Four profiles were defined: (1) people with diabetes who were frequent users, (2) people with diabetes who were infrequent users, (3) caregivers who were frequent users, and (4) caregivers who were infrequent users. Frequent users were defined as individuals who used the app 6 or more times during the month-long study period, whereas infrequent users were those who used the app fewer than 6 times that month.

Eligible participants were approached via 2 strategies. First, potential participants who had already finished using the app at home were contacted by telephone and invited to an interview. Second, potential participants who were invited to start using the app at home were also invited to participate in this study. For this option, researchers visited health care facilities and invited individuals in waiting rooms to participate. When approached, potential participants received information on the study objectives, conditions of participation, and voluntary nature of participation.

Study participants included people with diabetes and caregivers who had participated in the at-home use of the app to capture foot images. The demonstration of app interface and standard image capture is provided in Multimedia Appendix 1. The caregivers interviewed were not the caregivers of the people with diabetes interviewed in this qualitative sample, but instead they were the caregivers of other patients enrolled in the same parent study.

### Data Collection

In-depth individual interviews were conducted using a semistructured guide based on the COM-B subdomains. The interview guide in both the original Spanish and its English translation is provided in Multimedia Appendix 2. The guide explored initial experiences with the app, foot-monitoring habits, frequency and conditions of use, technological barriers, digital accessibility (device type, connectivity, and storage), social support, perceptions of diabetic foot risk, and suggestions for improving the tool’s design.

Questions were adapted according to participants’ profiles (people with diabetes or caregivers) while maintaining conceptual consistency across interviews. When feasible, participants were asked to describe or demonstrate how they accessed the app and captured foot photographs to identify practical usability challenges.

Most interviews were conducted in participants’ homes, and one interview was conducted remotely via videoconference based on participants’ preferences and availability. All interviews were conducted in private settings without the presence of third parties.

Interviews lasted approximately 45 to 60 minutes. All interviews were audio-recorded with prior authorization and transcribed verbatim. Repeat interviews were not conducted, and transcripts were not returned to participants. Data collection continued until meaning saturation was reached, which is defined as the point at which additional interviews no longer generated new insights or perspectives relevant to the analytic categories, indicating sufficient depth and coverage of the study objectives [19].

### Data Analysis

All interviews were anonymized using alphanumeric codes and analyzed in 2 sequential stages.

In the first stage, a deductive thematic analysis guided by the COM-B model and the TDF was conducted. The COM-B model provided the overarching behavioral structure, while the TDF offered an additional contextual layer for classifying determinants within each domain. Separate analytical matrices were constructed for people with diabetes and caregivers. Two researchers independently coded excerpts and classified them into COM-B domains and corresponding TDF subdomains, followed by consensus meetings to resolve discrepancies.

In the second stage, an inductive analysis was conducted within each domain to identify emergent cross-cutting themes among and between participant types. These themes were then compared between people with diabetes and caregivers to examine patterns shared between and distinct to the 2 groups. To do so, a second synthesis matrix was used to reorganize coded excerpts into themes while maintaining explicit links to the COM-B and TDF domains. Regular analytic meetings with the senior qualitative researcher and the broader research team were held to refine interpretations and strengthen analytic rigor. A sample matrix with domain and coding labels can be found in Multimedia Appendix 3.

Data management and analysis were manually conducted using structured Microsoft Excel matrices due to the team’s familiarity with this software. No specialized qualitative software was used. All quotes presented in this paper were originally collected in Spanish and have been translated into English for reporting purposes.

### Ethical Considerations

This study has received ethical approval from the following committees: the Institutional Ethics Committee of the Universidad Peruana Cayetano Heredia (reference number 210819), Lima, Peru, and the London School of Hygiene & Tropical Medicine Research Ethics Committee (reference number 30662), United Kingdom, as required for studies involving London School of Hygiene & Tropical Medicine–affiliated investigators and those funded by the UK Medical Research Council.

All participants provided written informed consent before enrollment. For qualitative interviews, additional consent was obtained for audio-recording. Participation was voluntary, and individuals could withdraw at any time without affecting their access to care.

To protect participants’ privacy and confidentiality, identifying information was removed from the study dataset, and each participant was assigned a unique study code. Study data were maintained in a secure spreadsheet accessible only to the research team. Participants who attended interviews in person received 20 Peruvian soles (US $5.89) to help cover transportation costs.

## Results

### General Findings

A total of 24 interviews were conducted: 14 with people with type 2 diabetes and 10 with caregivers. All caregivers interviewed were identified as female, were predominantly younger than 30 years, and were almost all informal family caregivers, except for one nonfamily caregiver (Table 1). In contrast, most people with diabetes were older than 50 years and had attained only up to a secondary education.

**Table 1.** Sociodemographic characteristics of interview participants, by role (caregiver vs patient).

Participant characteristic	Caregiver (N=10)	People with diabetes (N=14)
Gender		
Male	0	6
Female	10	8
Age group (y)		
≤30	6	0
31‐39	1	0
40‐49	2	2
50‐59	1	6
60‐69	0	5
70‐79	0	1
Highest education level attained^a^		
Primary	1	1
Secondary	5	11
Higher education^b^	4	2
History of ulceration	—^c^	6
History of amputation	—	4
History of deformity	—	7

a Primary and secondary education categories include participants who both completed and did not complete that level.

b Higher education in Peru includes postsecondary education at universities or nonuniversity higher education institutions (eg, technical or professional institutes), regardless of degree completion status.

c Not applicable.

Across transcripts, 239 excerpts were coded for people with diabetes and 332 for caregivers, using COM-B and TDF domains. These coded excerpts were inductively collapsed into general facilitators and barriers mapped to capability, opportunity, and motivation (Tables 2 and 3). Overall, although some themes predominantly aligned with a single COM-B domain, our analysis revealed substantial overlap across domains, with capability, opportunity, and motivation interacting dynamically to shape the app use. The following sections detail these cross-domain interactions, which are also visually summarized in Figure 2.

**Table 2.** Summary of facilitators and barriers across COM-B (capability, opportunity, motivation–behavior) domains identified among people with diabetes.

COM-B domain	General themes
Physical capability	
Facilitators	Independent ability to position and photograph the foot
Barriers	Limited mobility or difficulty reaching/visualizing the foot Physical fatigue or late-night exhaustion Need for assistance to complete positioning
Psychological capability	
Facilitators	Development of routines and reminder strategies Use of adaptive strategies (pillows, selfie sticks, and lamp positioning)
Barriers	Limited digital literacy Forgetfulness or inconsistent routine Initial confusion regarding installation and workflow
Physical opportunity	
Facilitators	Access to smartphones, internet connectivity, and adequate lighting Organized home spaces and established routines Ability to complete monitoring without traveling to hospital
Barriers	Unstable internet connectivity and device incompatibility App crashes, slow loading, and unclear submission confirmation Inadequate lighting or physical setup
Social opportunity	
Facilitators	Assistance from family members or household contacts External reminders from relatives Follow-up communication from health professionals
Barriers	Dependence on others for technical or physical assistance Inconsistent follow-up from field personnel during and after study completion
Automatic motivation	
Facilitators	Emotional reassurance from perceived monitoring Satisfaction from observing wound improvement Routine formation through repeated use
Barriers	No barrier identified
Reflective motivation	
Facilitators	Belief in early detection and prevention benefits Perceived convenience compared to hospital visits Commitment to research participation and helping others
Barrier	Doubt about whether photos were reviewed or useful Participation primarily driven by obligation rather than perceived personal value

**Table 3.** Summary of facilitators and barriers across COM-B (capability, opportunity, motivation–behavior) domains identified among caregivers.

COM-B domain	General themes
Physical capability	
Facilitators	Physical ability to position, lift, and assist people with diabetes enabled photo-taking despite mobility limitations of the person with diabetes Physical capability is strengthened through repetition and routine performance
Barriers	Physical fatigue from caregiving load, work, or illness reduced consistency of app use Patient mobility limitations required assistance for task completion
Psychological capability	
Facilitators	Knowledge of diabetic foot risk increased preparedness and engagement Caregivers demonstrated higher digital literacy than patients Caregivers exercised behavioral regulation and sought out answers themselves
Barriers	Memory, lapses, attention, and cognitive overload Initial confusion regarding installation of the app and workflow
Physical opportunity	
Facilitators	Access to smartphones, internet connectivity, and adequate lighting Organized home spaces and established routines Remote monitoring reduced need for hospital travel
Barriers	Unstable internet connectivity and device incompatibility App crashes, slow loading, and unclear submission of pictures (lack of confirmation) Time constraints due to work and caregiving responsibilities Inadequate lighting or physical setup
Social opportunity	
Facilitators	Family collaboration and task-sharing among relatives Follow-up and accountability from field personnel Patient reminders and mutual reinforcement within the household
Barriers	Limited or inconsistent family support Discontinuation of follow-up from field personnel
Automatic motivation	
Facilitators	Emotional reassurance from perceived monitoring Satisfaction from observing wound improvement Fear of complications (eg, amputation) prompting vigilance Routine formation through repeated use
Barriers	Frustration and stress from technical failures
Reflective motivation	
Facilitators	Belief in early detection and prevention benefits Perceived convenience compared to hospital visits Commitment to research participation and helping others
Barrier	Doubt about whether photos were reviewed or useful

**Figure 2.**
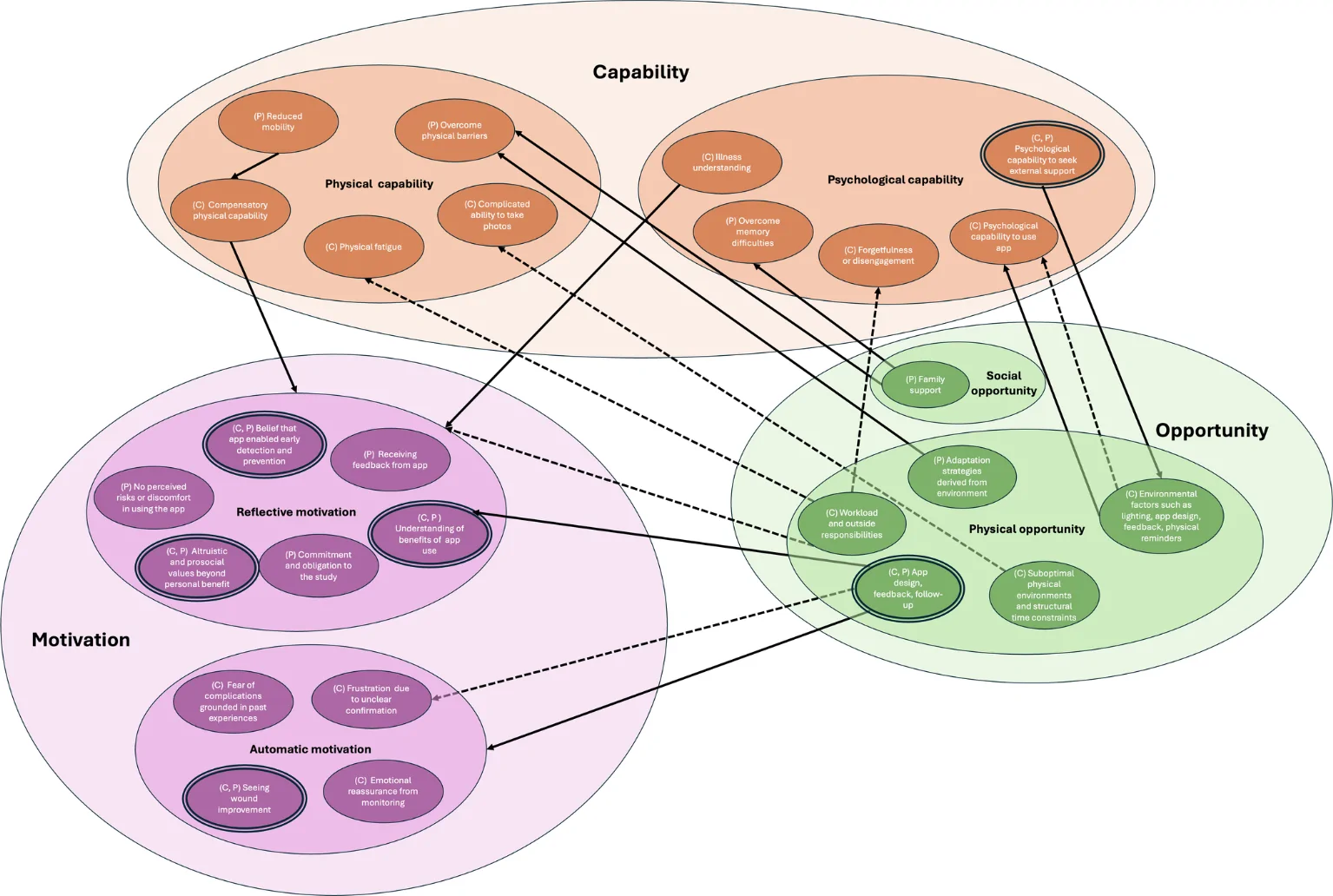
Cross-domain interactions that influence the sustained use of the app among people with diabetes and caregivers. This conceptual model illustrates how capability (purple), opportunity (green), and motivation (orange) domains interact to shape engagement with the intervention. Nodes labeled (P) reflect patient-specific influences, (C) reflect caregiver-specific influences, and double-lined (C and P) reflect shared determinants. Solid arrows indicate positive influences, whereas dashed arrows represent negative relationships.

### Capability

The biggest difference observed between caregivers and people with diabetes was their capabilities. When people with diabetes had reduced mobility, caregivers’ physical capabilities acted as a compensatory facilitator, enabling task completion through lifting, positioning, or adapting the environment (TDF domain: physical skills). A person’s lack of physical capability served as a motivating factor for caregivers to use the app.

*Only I could handle the app, position my mom […] I adjust her, and then I just take the photo*.

[Caregiver, female, 23 years old]

Still, even when caregivers had additional skills required to use the app, physical fatigue from the caregiving load, work, or illness served as a barrier, reducing consistency and delaying task completion. In this way, people with diabetes and caregivers alike experienced physical capability barriers, but for different reasons.

*I would get home at 10 at night… I was already sleepy, tired, and then it would be tomorrow already, and I wouldn’t take it anymore*.

[Caregiver, female, 50 years old]

*Well, what happens is that since it’s the feet, it’s like I’m a bit overweight, but that depends on me, not on the application*.

[Patient, female, 50 years old]

Interestingly, some people with diabetes used adaptation strategies to overcome these limitations, such as using a pillow or a selfie stick or taking a photo during the day to take advantage of the lighting (TDF domain: behavioral regulation).

*On the second day it was difficult for me because I had to take the photos by myself and for [the app] to take the photo […] I had to put pillows, then I used the little stick to take photos*.

[Patient, female, 41 years old]

Many people with diabetes were psychologically capable of using the app and acquired this ability through practice and other strategies, such as setting reminders and developing routines for taking pictures (TDF domain: behavioral regulation). Caregivers also experienced initial confusion with the app, which was resolved through practice, demonstration, repetition, or troubleshooting (TDF domain: memory, attention, and decision processes).

*The first few days, yes, two or three days I would get confused [with the app] […] But afterward it became easier. Afterward you get the…the trick. The trick, as they say, right? And then normally, you send [the photo]*.

[Patient, male, 51 years old]

*At first it was a little difficult, but after that…I got the hang of it*.

[Caregiver, female, 25 years old]

Additionally, caregivers seemed to better understand the seriousness of diabetic foot complications (eg, infection and amputation) and the purpose of the app as preventive monitoring, which made them more motivated and cognitively prepared to engage (TDF domain: knowledge). This understanding reframed the task from “taking photos” to preventing future harm, which supported sustained attention and follow-through.

*I am aware that a person with diabetes… can become infected and progress to the point that they can amputate it*.

[Caregiver, female, 25 years old]

### Opportunity

Both caregivers and people with diabetes who had the opportunity to perform foot monitoring depended on having access to the internet, smartphones, lighting, usable home spaces, and physical reminders. For caregivers, these environmental conditions enabled the behavior and interacted with capability: poor lighting or device limitations increased physical strain and cognitive effort, while adequate setups reduced both (TDF domain: environmental context and resources). People with diabetes who had these adequate environmental conditions were able to perform the behavior consistently, while those who did not were unable to complete the task without adaptation (TDF domain: environmental context and resources).

*We always looked for the room with good lighting.*

[Caregiver, female, 21 years old]

*Well, in my case, I have internet at home […] I never had any problems*.

[Patient, female, 50 years old]

*The first day, two days, yes, it took us a while because sometimes with the light my foot would come out crooked, then little by little I learned the position […] and at night I would turn on my lamp so that it would focus on me […] sometimes the light would reflect and I would say, no, the light is positioned wrong, I remember*.

[Patient, male, 65 years old]

In terms of app performance, people with diabetes reported that although the app was often easy to use due to its intuitive interface, structural barriers—including device incompatibility, inability to verify whether photos had been successfully sent, and the need to repeatedly reenter the app workflow—created disruptions in their opportunity to complete the task consistently. For caregivers, clear visual design and feedback enabled opportunity and motivation by making photo-taking more feasible. Conversely, slow loading, crashes, unclear confirmation, and device incompatibility disrupted opportunity, decreased motivation, and simultaneously increased cognitive uncertainty and frustration.

*I had to buy another cellphone, because the one I had wouldn’t go in anymore […] No, it wouldn’t allow it*.

[Patient, male, 51 years old]

*It would freeze […] I had to take the photo again*.

[Caregiver, female, 27 years old]

Caregivers frequently faced structural time constraints due to work, caregiving for others, unstable schedules, and an overall lack of routine. These factors limited physical opportunities to perform the task, even when motivation and capability were present. These constraints also produced fatigue, forgetfulness, or disengagement.

*[…] I just don’t have enough time to stay and check. Even though it doesn’t take long, honestly, I don’t have a set schedule — sometimes I work in the morning, sometimes in the afternoon, sometimes at night*.

[Caregiver, female, 23 years old]

Social support was a dominant structural enabler of participation for people with diabetes and caregivers, but the underlying mechanisms differed. Many people with diabetes depended on children, siblings, or even building staff to take photos, enter login credentials, or troubleshoot (TDF domain: social influences). For several people with diabetes, independent use was not feasible without assistance due to physical positioning challenges, digital literacy limitations, or difficulty visualizing their own feet.

*Sometimes my husband [took the photographs]. I told him, move here or he would take about 10 photos and then I would choose*.

[Patient, female, 41 years old]

*It became difficult for me so I looked for the security guard to help me take the photo, nothing more. I did the programming and he would just take the photo.*

[Patient, male, 65 years old]

In contrast, caregivers’ reliance on social support did not stem from a lack of capability. Caregivers were generally able to perform the task independently; however, sustaining participation required coordination within the household. Family collaboration functioned as task-sharing rather than substitution, with relatives rotating through roles such as taking photos, providing reminders, or assisting when the primary caregiver was unavailable. Follow-up by health staff similarly reinforced continuity by serving as an external structure of accountability.

*And if I couldn’t, like I’m telling you, if I couldn’t be there one or two days, my son was here with him all day. And he stayed like that. He would watch how I [took the photos], and he had already memorized it too*.

[Caregiver, female, 42 years old]

*The same doctor, the nurse, kept communicating that there was progress, and afterward she told me: ‘Okay, that’s fine, up to here,’ and we didn’t send any more photos.*

[Caregiver, female, 23 years old]

### Motivation

By far, the most common motivator mentioned among caregivers and people with diabetes was a belief about the consequences of app use, whether that was a belief in the potential for prevention of DFU or a belief in the convenience of taking care of your feet at home rather than in a hospital (TDF domain: beliefs about consequences). Caregivers were motivated when they believed that the app enabled early detection and prevention, reframing the behavior as proactive rather than reactive. Motivation to use the app for people with diabetes arose from personal benefits and the detection of health problems that were not initially perceived.

*If a lesion were to suddenly appear and you let me know, we can act sooner. That way we prevent it*.

[Caregiver, female, 32 years old]

…*Because in the long run the benefit will be for us, the diagnoses will be for us, not for the person who received the photos, but for ourselves, so that’s why I did it*.

[Patient, female, 53 years old]

*No, no, because I dedicate 10 minutes and that’s it, I can dedicate more hours to TikTok, to Facebook, and this is something useful, and you learn. If they send me, put this on, you can eat this, I don’t know, more information about my disease, great, I read it, because I read*.

[Patient, female, 50 years old]

Among people with diabetes, some participated in photo-taking out of commitment to the research study, which ties into the “reflective motivation” TDF domain of social/professional role or identity. In these cases, the use of the app exclusively arises out of an obligation and a belief in the value of their commitment (TDF domain: intentions). However, some still recognized the app’s usefulness, which helped reinforce more consistent use through curiosity, self-care, and control over their health.

*More than anything because I had made a commitment to the young lady (research team member), I had committed to helping. So with the study, more than anything it was for that reason, because I am a person that if I commit to something it is because I am going to try to do it, to fulfill it*.

[Patient, female, 53 years old]

An unexpected motivator that emerged for both caregivers and people with diabetes was a desire to help others or altruism. Some caregivers framed their use of the app not as a means to benefit their own family members, but as a way to contribute to research, support other people with diabetes, and improve care for the broader community (TDF domain: social/professional role and identity). This altruistic framing sustained engagement even when direct personal benefits were limited or uncertain. People with diabetes expressed a desire to help other people with diabetes and share experiences even when they felt exhausted or faced difficulties in daily life.

*Why? Because you’re going to help someone else. Well, that’s what moved me, that it is to help other people to see. I think that’s why it motivates you to do it, that it’s help for others, right? To tell you about your experience […] I liked it because it is to help other people*.

[Patient, female, 41 years old]

*I’m not doing it only for my mom… but because I see that it’s an app that is going to help other people*.

[Caregiver, female, 25 years old]

For people with diabetes, automatic motivation interpreted as reinforcement functioned as a key facilitator. Receiving feedback on submitted photos or observing visible wound improvement generated satisfaction, reassurance, and increased confidence in their ability to manage their condition (TDF domain: reinforcement). These positive emotional responses and sense of relief reinforced routine use and strengthened habit formation over time. Similarly, caregivers described reinforcement through visible clinical progress or confirmation from health care staff. Observing improvement in the foot or receiving acknowledgment that images had been reviewed produced pride and relief, reinforcing continued monitoring behavior.

*Now [the application] was useful because they have seen that I am well and they have monitored me and I like being well.*

[Patient, female, 50 years old]

*Sometimes it felt good […] even with the wound. Whenever it improves, you feel happy: ‘Look at how the photo looks!’ Comparing the first photo with the last is always nice because it’s the change—you can see the improvement*.

[Caregiver, female, 23 years old]

Emotion-driven vigilance was also prominent, particularly among caregivers (TDF domain: emotion). Fear of complications, especially amputation, served as a powerful emotional driver that heightened attention and sustained adherence to monitoring. This fear was often grounded in prior experiences with severe diabetic complications within the family or community.

*That’s what scares me—that it could get to that point… that they might have to amputate the foot*.

[Caregiver, female, 21 years old]

In one particular instance involving a caregiver, frustration and uncertainty about the app undermined automatic motivation. Technical failures, unclear confirmation of image receipt, and ambiguity about whether photos were being reviewed generated stress, disengagement, and overall demotivation. Ultimately, these negative emotional responses led to the permanent abandonment of the prototype app by this caregiver.

*That stressed me out… and that made me quit [the app]*.

[Caregiver, female, 21 years old]

## Discussion

### Principal Findings

In this study, we explored behavioral influences on the use of an mHealth app for diabetic foot care in Peru. After our analysis with the COM-B model and TDF, we found that the sustained use of the app for the detection of DFUs and preulcerative lesions was not always determined by a single behavioral domain but rather by interactions among capability, opportunity, and motivation among people with diabetes and caregivers. These interactions were strongly influenced by conditions across the social, technological, and health care systems in Peru, highlighting the importance of incorporating stakeholder experiences and behaviors from the earliest stages of implementation, including the algorithmic development of an mHealth intervention.

When discussing individual domains, physical capability barriers were particularly evident among people with diabetes who had mobility limitations, while caregivers frequently compensated by positioning the foot to take pictures or by making environmental adjustments, such as modifying light and room settings. Opportunity was strongly shaped by structural and technological conditions, including device compatibility, internet access, and home environments, which in turn conditioned how these behaviors could be carried out. Meanwhile, motivation was largely driven by beliefs about the preventive value of the intervention, emotional responses to first-hand experiences with DFU, and reassurance that health professionals were monitoring submitted images. Other motivational influences included altruistic reasons for sustaining engagement with the app, whereas uncertainty about whether images were being received could undermine motivation. Together, these results highlight that engagement with our digital self-care intervention stems from various behavioral interactions rather than only isolated determinants.

A key contribution of this study is the identification of people with diabetes and their caregivers as a behavioral unit or dyad in the use of digital health technologies. While many mHealth interventions conceptualize self-monitoring as an empowering, individual activity [20-22], our findings show that the routine use often depended on collaboration within the household. In several multigenerational households, family members, mostly children or grandchildren, rotated responsibilities when the primary caregiver was unavailable. Previous research on remote monitoring technologies for diabetes similarly suggests that caregiver involvement can facilitate adherence and improve clinical outcomes, particularly among older adults and individuals with lower digital literacy [23].

In our sample, this dynamic was also reflected in the demographic profile of participants. People with diabetes were substantially older than caregivers (median age of 54 y vs 28.5 y) and had generally attained lower levels of formal education. These differences may have contributed to variations in both familiarity with smartphone interfaces and illness-related knowledge, as reported in other studies [24,25]. Although smartphone ownership is widespread in Peru [26-28], several people with diabetes described challenges related to positioning the foot for photographs, reading small text, or navigating app steps. Despite these physical struggles, people with diabetes whom we interviewed still demonstrated adequate psychological capability to understand the purpose of the intervention and even used adaptation strategies to continue using the app. In these cases, caregivers did not replace the decision-making capacity of people with diabetes but instead bridged physical and informational gaps associated with performing the task. By supporting the practical and cognitive demands associated with technology use, caregivers enabled participation among people with diabetes who might otherwise be excluded from technological interventions [29,30].

Building on the concept of a dyadic relationship between people with diabetes and caregivers, the involvement of caregivers also introduces additional complexity. While digital technologies are often promoted as tools that reduce caregiver burden, evidence suggests that they may simultaneously introduce new responsibilities and stressors [31]. Studies examining the impact of assistive technologies on informal caregivers have found that while these tools can improve communication, monitoring, and perceived safety, they may also generate frustration when technical difficulties arise or when the technology requires additional time and attention to operate [31].

Importantly, the caregiving role itself is highly gendered, which may intersect with the age-related differences in digital literacy that we observed. In our sample, all caregivers interviewed were women, a pattern that reflects broader trends documented in the literature, where informal caregiving responsibilities disproportionately fall on female family members, particularly in Latin American contexts [31-33]. Although we used a purposive sampling strategy to capture a diverse range of app-use profiles (eg, frequent and infrequent users) as well as variation in gender, our caregiver sample did not include male caregivers. As a result, our findings may not fully capture the behaviors and interactions between people with diabetes and male caregivers. This gendered pattern is important because research examining the use of technology among informal female caregivers has shown that, while digital tools may support caregiving tasks, they can also add to existing responsibilities and contribute to declines in caregivers’ physical and mental well-being over time [32]. This is especially relevant in settings where caregiving responsibilities are often balanced alongside employment, household duties, or educational commitments.

Similar patterns emerged in our investigation. Caregivers frequently described time constraints, competing responsibilities, and fatigue that limited their ability to perform monitoring tasks consistently. However, most participants did not describe the app itself as a direct burden. Instead, the challenges they described were primarily related to the broader demands of caregiving and daily life, such as work or school obligations. Only one caregiver explicitly reported disengaging from the app due to frustration with technical difficulties, which were also reported by Holthe et al [34] as a source of stress for caregivers working with assistive technology. These instances suggest that even relatively simple digital monitoring interventions can introduce new routines and obligations that must be considered during intervention design.

Another important finding relates to participants’ strong preference for follow-up and reassurance from health care professionals. Both caregivers and people with diabetes described feeling more motivated to use the app when they believed that a clinician was reviewing their images or monitoring their progress. Previous studies of digital interventions have similarly shown that remote monitoring had a reassuring effect on people with diabetes and allowed them to share the responsibility of their health care decisions with others [21,29]. Interestingly, although these studies involved clinicians directly reviewing patients’ results, our mHealth app did not include clinicians’ review of submitted images for diagnosis. Instead, follow-up only occurred if any lesions were found or if participants had discontinued the app use. During interviews, participants often interpreted this follow-up communication and the submission of photos as physicians reviewing their images, even though this was not outlined in the participant information and consent forms. This finding in particular has been considered for future iterations of the app within the Foot Selfie study. Future versions of the app are set to provide clearer feedback confirming whether images were successfully submitted and clarifying that diagnostic feedback is AI-generated rather than determined by a physician.

This demonstrated preference for follow-up may be related to the broader preference for clinician-guided care and paternalism described in Peru and other contexts outside of Western medicine [35-37]. Although paternalism is often defined as physicians making decisions for people with diabetes without regard for patient autonomy, in this cultural context, the desire for authority may instead reflect how people with diabetes interpret care and attention from health care professionals, as seen in other qualitative studies [38]. In other words, follow-up appeared to signal concern and commitment from clinicians, which in turn increased the perceived value of the app among participants. This interpretation is consistent with a preliminary study from our research group, in which both the intervention and control arms of a DFU prevention trial remained more engaged than expected, suggesting that regular contact and follow-up may itself influence self-care behaviors [39]. Brandt et al [40] reinforced this finding and found that participants were not as motivated or adherent with a mobile self-help intervention in Peru when they were left without the support of a nurse. Taken together, these findings suggest that sustained engagement in our study may have been driven in part by participants’ perception that a health professional was monitoring their submissions, rather than by the digital intervention alone.

Participants’ desire for continuity of care in this study raises questions about the sustainability of implementing such an intervention if its primary goal is to address gaps in access to care and promote preventive self-management. The feasibility of having health care professionals regularly reviewing submissions is limited, particularly in a health care system such as Peru’s that is already resource-constrained and where people with diabetes often experience long wait times to access specialist care [37]. In this context, AI-assisted identification of preulcerative lesions may provide a form of timely preliminary review, helping prioritize clinicians’ attention for participants who need further evaluation. As a whole, future implementation of the app may be more effective if it incorporates other actors, including caregivers, nonphysician health personnel, and AI-assisted triage, to support continuity without relying solely on regular clinician review.

### Limitations

Although the use of the COM-B model combined with the TDF provided a structured approach to understanding behavioral determinants of app use, the interpretation of qualitative interview data remains inherently subjective. The analytic framework may also have shaped how themes were identified and interpreted. To enhance analytic rigor, regular meetings were held within the research team to review coding decisions and discuss interpretations. Because the analysis relied on predefined theoretical domains, it is possible that certain factors influencing behavior were interpreted primarily through the lens of COM-B and TDF, despite regular team-based reviews of coding decisions.

During interviews, participants may have exhibited social desirability bias by portraying the app more favorably, even though the interviewers were not previously known to them. Despite sampling participants who were not frequent users of the app, it is still possible that individuals with negative experiences might not have fully disclosed them due to this bias. To mitigate this, we conducted semistructured interviews, and interviewers were trained to build trust, maintain confidentiality, and remain neutral. Additionally, the caregivers we interviewed were not the caregivers of the people with diabetes included in our sample, limiting our ability to directly assess concordance or discordance in beliefs within patient-caregiver dyads.

### Implications

Importantly, the app used during the study was an early prototype developed for algorithmic training. While participants reported several technical challenges that at times affected engagement, these experiences provided critical insight into real-world usability and contextual barriers. Incorporating user feedback from this early stage allowed for refinement of the app through subsequent cocreation workshops with stakeholders and engineers, resulting in an improved version better aligned with the needs of people with diabetes in Peru.

Our findings also imply that app design should prioritize usability across diverse technological contexts, including compatibility with older devices and clear feedback confirming successful data submission. Alongside this, interventions should explicitly consider caregivers as key actors in digital self-monitoring programs, particularly for older adults or individuals with limited mobility. Training materials, reminders, and communication features may therefore need to address both people with diabetes and caregivers. Prior research examining dyadic relationships in chronic heart failure care similarly highlights how interactions between people with heart failure and caregivers can influence disease management behaviors, suggesting that approaches engaging both members of the caregiving dyad may strengthen the effectiveness of self-management interventions [25]. Furthermore, since the use of mHealth tools can, at times, be a burden for caregivers, informal task-sharing among other household members should be considered for implementation. Finally, the strong desire for professional follow-up suggests that future versions of the intervention may benefit from hybrid models combining self-monitoring with targeted human support. The AI-assisted identification of concerning findings could help guide review by nurses or other nonphysician health professionals, with escalation when needed, preserving the preventive focus of the intervention while supporting realistic continuity of care.

### Conclusions

As mHealth interventions for diabetic foot self-care continue to expand, our study demonstrates how examining behavioral interactions between people with diabetes and caregivers can inform the development of person-centered mobile interventions. We found that sustained engagement with the app for diabetic foot self-care emerges from dynamic interactions between capability, opportunity, and motivation rather than any single behavioral determinant for participants. In this study, the app frequently depended on collaboration between people with diabetes and various household caregivers or family members, who together functioned as a behavioral unit that jointly enabled participation in digital monitoring. This central finding underscores the importance of designing digital health interventions that account for household caregiving dynamics, technological constraints, and user perceptions of clinical support. As these insights are incorporated into future versions of the app, considering behavioral and contextual factors may improve the implementation and long-term adoption of mHealth tools aimed at preventing diabetic foot complications in Peru and other low- and middle-income settings, as well as within health care organizations.

## Supplementary material

10.2196/101195 Multimedia Appendix 1 Foot Selfie mobile app used for foot image capture.

10.2196/101195 Multimedia Appendix 2 Semistructured interview guide for people with diabetes and caregivers regarding the use and accessibility of the Foot Selfie mobile app in both original Spanish and its English translation.

10.2196/101195 Multimedia Appendix 3 A sample matrix with domain and coding labels for COM-B (capability, opportunity, motivation–behavior) and Theoretical Domains Framework (TDF) transcript analysis.

## Abbreviations

APK: Android package kit
COM-B: capability, opportunity, motivation–behavior
COREQ: Consolidated Criteria for Reporting Qualitative Research
DFU: diabetic foot ulcer
mHealth: mobile health
TDF: Theoretical Domains Framework
