# Supplementary material for: Behavioral Interactions Shaping the Use of a Mobile Health Intervention for Diabetic Foot Self-Care Among People With Diabetes and Caregivers: COM-B and TDF Qualitative Analysis
Source: JMIR Mhealth Uhealth. 2026 Nov 10;14:e101195. doi: 10.2196/101195 (PMC13750630; doi:10.2196/101195)
Supplement: Supplementary file 1 [file mhealth-v14-e101195-s001.docx]

**Supplementary Figure S1. Foot Selfie mobile application used for foot image capture.**

Screenshots of the Foot Selfie mobile application used during the study. Panels illustrate the login interface (A), standardized plantar image capture with color calibration marker (B).

**(A)** Application login interface screen	**(B)** Standardized plantar image capture

with color calibration marker
