# Supplementary material for: Behavioral Interactions Shaping the Use of a Mobile Health Intervention for Diabetic Foot Self-Care Among People With Diabetes and Caregivers: COM-B and TDF Qualitative Analysis
Source: JMIR Mhealth Uhealth. 2026 Nov 10;14:e101195. doi: 10.2196/101195 (PMC13750630; doi:10.2196/101195)
Supplement: Supplementary file 2 [file mhealth-v14-e101195-s002.docx]

**Appendix 2. Semi-Structured Interview Guide for People With Diabetes and Caregivers Regarding Use and Accessibility of the Foot Selfie Mobile Application in both original Spanish and an English translation.**

RELATIONSHIP WITH FOOT CARE AND DIABETIC FOOT

1. What are your thoughts about your feet?
2. Do you usually check your feet?
   a. If “Yes,” explore: How do you check them? How often? Does someone help you or accompany you? Why do you check them? *(Explore whether foot checks are part of an existing routine, such as bathing, caregiving, or another routine.*)
   b. If “No,” explore: What would you need in order to start checking your feet? At what point in your day could you incorporate a foot check? How would you do it using the application? How would you do it without the application?
3. What do you know about diabetic foot? How would you like information or follow-up to be provided to help prevent diabetic foot complications?
4. Have you ever had diabetic foot complications? *Explore*: What treatment did you receive? (This helps us understand the severity of the participant’s previous diabetic foot condition.)

CAPABILITY AND OPPORTUNITY. Explore internal and external factors that facilitate or hinder the behavior.

5. Tell me, what was your first thought when you heard about this application? *(Explore: Concerns about personal data or privacy, perceptions that the application might be expensive or require payment, difficulties related to the use of or access to digital technologies, lack of interest, etc.)*
6. What was your experience the first time you used the application? How did you feel, and why? *(Explore: Feelings such as curiosity, frustration, confidence in one’s ability to use the application, etc.) [Motivation]*
7. How often did you use the application at home to take photographs of your feet? What influenced how often you used it?
8. How did you remember that you needed to use the application? What else could help remind you to use it? [Motivation]
9. Tell me, who took the photograph?
   a. If someone else took the photograph: Tell me about the last time someone took the photograph for you. Do you remember what steps they followed?
   b. If the participant took the photograph: Tell me about the last time you took photographs of your feet using the application.

Explore the steps followed: Could you please show me how you open the application and take the photographs? *(Document the steps in relation to the intended application workflow.)*

10. While using the application, what did you find easiest? Did you encounter any difficulties? What were they, and how did you address them?

Explore: Design-related aspects such as font size, colors, and the application name, as well as difficulties installing the application, accessing it, taking photographs, or using it because of factors such as phone type, internet connectivity, alerts, etc.

MOTIVATION. Explore why participants engage in the behavior.

11. Some people did not use the application. What do you think might be the reasons for this?
12. Earlier, you told me what you thought about the mobile application before using it. Now that you have used it, what do you think about it?
13. Did you feel that this application was useful to you? In what ways? *(Explore: Greater confidence or ability to manage one’s care, organization of caregiving responsibilities within the family, digital knowledge or skills, emotions, etc.)*
14. Did you feel that you gave up or lost anything by using the application? If so, what? *(Explore: Time, money, emotional burden, etc.)*
15. Would you recommend this application to friends or family members who have diabetes? Why or why not?
16. If you wanted to promote this application as a way to help prevent diabetic foot complications, how would you introduce or share it within your family? What about within your community or neighborhood?

FUTURE USE OF THE APPLICATION

Imagine that there is a second version of the application we have been discussing, designed to help you care for your feet and prevent diabetic foot complications.

17. What would need to happen in your life for you to be able to use it? *(Explore: How similar or different would using the application be from something you already do?)*
18. When do you think you could use an application to help care for your feet? At what point in your daily routine would you use it?
19. How often do you think you could use it in a typical month? Why?
20. Where could you use it? Why?
21. Whose help or support, if anyone, would you need in order to use it?
22. If you had the opportunity to design this application with us, what would you suggest changing or improving so that it would genuinely help you?
23. How would you like the application and how to use it to be explained to you?

APPLICATION NAME

24. We are looking for a name for a mobile application designed to support the prevention of diabetic foot complications. If you could name the application, what would you call it? Why?
25. What comes to mind when you hear the name “Foot Selfie”? Do you think this name reflects or relates to the purpose of the proposed application?

**Original Spanish Guide**

RELACIÓN CON LOS PIES Y EL PIE DIABÉTICO

1. ¿Qué piensa de sus pies?
2. ¿Usted normalmente revisa sus pies?
a. En caso responda “Sí”. *Explorar:* ¿cómo lo hace?, ¿cada cuánto tiempo?, ¿lo hace acompañado? ¿Por qué lo hace? *(Conocer si forma parte de alguna rutina de baño, de cuidado del familiar, entre otros)*.
b. En caso responda “No”. *Explorar:* ¿qué necesitaría para hacerlo? ¿En qué momento de su día podría incluir la revisión de sus pies? ¿Cómo lo haría con la aplicación?, ¿cómo lo haría sin la aplicación?
3. ¿Qué conoce sobre el pie diabético? ¿Cómo le gustaría que le expliquen o le hagan seguimiento para prevenir este diagnóstico?
4. ¿Ha tenido usted pie diabético? Explorar qué tratamiento recibió (esto nos ayuda a entender el grado del pie diabético).

CAPACIDAD Y OPORTUNIDAD. Explorar los factores internos y externos que facilitan o dificultan el comportamiento.

5. Cuénteme, ¿qué pensó por primera vez cuando escuchó de esta aplicación? *(Explorar desconfianza por datos personales, ideas de que es cara o podría costar, complejidad por uso o acceso a TIC, desinterés, etc.).*
6. ¿Cómo fue la primera vez que usó esta aplicación?, ¿cómo se sintió?, ¿por qué? *(Explorar sensación de curiosidad, frustración, competencia, etc.)* *[Motivación]*
7. ¿Con qué frecuencia utilizó la aplicación para la toma de fotografías de pies en su hogar? ¿A qué se debió ello?
8. ¿Cómo recordaba que tenía que usar la aplicación? ¿Qué otras cosas la/lo ayudarían a recordar que tiene que usarla? *[Motivación]*
9. Cuénteme, ¿quién tomó la fotografía?
   a. *En caso la foto fue tomada por alguien más:* ¿Cómo fue la última vez que le tomaron la fotografía? ¿Recuerda qué pasos siguió?
   b. *En caso el participante haya tomado la fotografía:* ¿Cómo fue la última vez que usted tomó fotografías de los pies usando la app?

Explorar qué pasos siguió: Podría enseñarme, por favor, cómo ingresar y tomar fotos. *(Tomar nota de pasos según uso establecido)*

10. Durante su uso, ¿qué fue lo más fácil? ¿Encontró dificultades?, ¿cuáles fueron? ¿cómo las resolvió?

Explorar desde temas de diseño como tamaño de letra, colores, nombre de la app, etc., así como problemas para instalar la app o acceder y tomar fotos, accesibilidad relacionada al tipo de celular, conexión a internet, alertas, etc.

MOTIVACIÓN. Explorar por qué realizan el comportamiento.

11. Hay personas que no han usado la aplicación ¿cuáles cree que son las razones para ello?
12. Hace un momento me comentó qué pensó antes de usar la aplicación móvil, ahora tras su uso, ¿qué piensa de la aplicación?
13. ¿Sintió que esta aplicación le ha sido útil? ¿Cómo? *(Explorar empoderamiento, organización familiar para distribuir cuidados, conocimiento digital, emociones, etc.)*
14. ¿Sintió que perdió algo por usar la aplicación? ¿Qué? *(Explorar tiempo, dinero, emociones, etc.)*
15. ¿Recomendaría esta app a sus amigos o familiares que tienen diabetes? ¿Por qué?
16. Si tuvieras que difundir esta aplicación para prevenir el pie diabético, ¿cómo lo harías en tu familia?, ¿en tu comunidad/vecindario?

USO FUTURO DE LA APLICACIÓN

Imaginemos que existe una versión dos de la aplicación que hemos venido conversando y que sirve para ayudarte en el cuidado de tus pies y prevención de pie diabético.

17. ¿Qué necesitarías que suceda en tu vida para que puedas utilizarla? *(Explorar: ¿qué tanto sería diferente o común a algo que ya estás haciendo?)*
18. ¿Cuándo crees que podrías usar una aplicación para el cuidado de tus pies? ¿En qué momento de tu día a día?
19. ¿Qué tan seguido al mes podrías usarlo? ¿Porqué?
20. ¿Dónde podrías usarla? ¿Porqué?
21. ¿A quiénes necesitarías para usarla?
22. Si tuvieras la oportunidad de diseñar esta aplicación con nosotros, ¿qué nos sugerirías para mejorarla y que realmente te ayude?
23. ¿Cómo te gustaría que te expliquen sobre el funcionamiento de la aplicación?

NOMBRE DE LA APLICACIÓN

24. Estamos buscando un nombre para una aplicación móvil que promueva la prevención del pie diabético. Si pudieras ponerle un nombre, ¿cuál sería? ¿Por qué?
25. ¿En qué piensas cuando escuchas el nombre Foot Selfie? ¿Le parece que guarda alguna relación con la aplicación propuesta?
